# Insulin Resistance and Suicidal Behaviors: Insights into Mood Disorders

**DOI:** 10.1192/j.eurpsy.2025.439

**Published:** 2025-08-26

**Authors:** L. Raffaelli, E. Mazza, C. Gaggi, C. Lorenzi, F. Benedetti

**Affiliations:** 1 IRCCS Ospedale San Raffaele, Milan, Italy

## Abstract

**Introduction:**

Compared to the general population, mood disorders (MD) patients show an increased risk of developing type II diabetes and obesity, which are associated with changes in brain correlates and a worse clinical outcome^[1]^. According to the literature, MD patients with a dysregulated metabolic system are characterized by a reduction in white matter (WM) integrity, lower global functioning, and suicidal behaviors (SB) ^[2],[3],[4]^. However, little is known about the impact of early stages of metabolic dysregulation, namely insulin resistance (IR), in relation to the clinical course of MD^[1]^.

**Objectives:**

Therefore, the present study aims to investigate the effect of IR on WM integrity in MD patients with suicidal behaviors (s-BP, s-UP) compared to those without suicidal behaviors (ns-BP, ns-UP). Finally, we have hypothesized that obesity may be linked to SB through a biological pathway involving inflammatory and IR markers.

**Methods:**

Our sample was composed of 184 depressed patients (92 BP, 92 UP) who were assessed for SB via the Beck Suicidal Scale (BSS). Patients underwent 3T Magnetic Resonance imaging, and blood samples were collected to determine levels of insulin and glucose and blood cell counts. The Homeostatic Model Assessment for Insulin Resistance (HOMA) and systemic-immune-inflammation index (SII) were then computed. To investigate the effect of HOMA and SII on WM microstructure, we performed voxelwise DTI analyses: first, we tested whether the relation between HOMA, SII, and DTI measures differed between s-BP and ns-BP patients; then, post-hoc analyses were performed for analyzing the effect of HOMA, and SII separately in 40 s-BP and 52 ns-BP. The same analyses were replicated on 43 s-UP and 49 ns-UP. Moderated mediation analyses were performed with the macro PROCESS for SPSS.

**Results:**

The relationship between BMI and suicidal behaviors was fully serial mediated by SII and HOMA only in BP (b=0,031, 95% BCa CI [0.003, 0.088]). Specifically, we found that higher BMI was sequentially associated with increased SII and HOMA levels, ultimately leading to higher BSS scores. A significant interaction between s-BP and ns-BP was identified for the effect of (1) HOMA on mean diffusivity (MD), axial (AD), and radial diffusivity (RD). However, no significant interaction was found for the effect of IR and SII markers in UP. Performing the analyses separately in the two groups, s-BP showed (1) a negative widespread association between HOMA and FA, and a positive effect between HOMA and RD, AD, and MD. In ns-BP, no significant results were found.

**Conclusions:**

These findings may suggest that IR may play a key role in the biological pathway underlying suicidal behaviors in BP but not in UP. Therefore, metabolic system dysregulation should be taken into consideration during the treatment.

**Disclosure of Interest:**

None Declared

